# Sensitivity of *Neurospora crassa* to a Marine-Derived* Aspergillus tubingensis* Anhydride Exhibiting Antifungal Activity That Is Mediated by the MAS1 Protein

**DOI:** 10.3390/md12094713

**Published:** 2014-09-01

**Authors:** Liat Koch, Anat Lodin, Inbal Herold, Micha Ilan, Shmuel Carmeli, Oded Yarden

**Affiliations:** 1 Department of Plant Pathology and Microbiology, The R.H. Smith Faculty of Agriculture, Food and Environment, The Hebrew University of Jerusalem, Rehovot 76100, Israel; E-Mails: liat.koch@mail.huji.ac.il (L.K.); inbiherold@gmail.com (I.H.); 2 School of Chemistry, Raymond and Beverly Sackler Faculty of Exact Sciences, Tel Aviv 69978, Israel; E-Mails: anatlodin@gmail.com (A.L.); carmeli@post.tau.ac.il (S.C.); 3 Department of Zoology, George S. Wise Faculty of Life Sciences, Tel Aviv University, Tel Aviv 69978, Israel; E-Mail: Milan@post.tau.ac.il

**Keywords:** natural products, antifungal, *Aspergillustubingensis*, *Neurospora crassa*, cell wall, chitin synthase

## Abstract

The fungus *Aspergillus* *tubingensis* (strain OY907) was isolated from the Mediterranean marine sponge *Ircinia variabilis*. Extracellular extracts produced by this strain were found to inhibit the growth of several fungi. Among the secreted extract components, a novel anhydride metabolite, tubingenoic anhydride A (**1**) as well as the known 2-carboxymethyl-3-hexylmaleic acid anhydride, asperic acid, and campyrone A and C were purified and their structure elucidated. Compound **1** and 2-carboxymethyl-3-hexylmaleic acid anhydride inhibited *Neurospora crassa* growth (MIC = 330 and 207 μM, respectively) and affected hyphal morphology. We produced a *N. crassa* mutant exhibiting tolerance to **1** and found that a yet-uncharacterized gene, designated *mas-1*, whose product is a cytosolic protein, confers sensitivity to this compound. The ∆*mas-1* strain showed increased tolerance to sublethal concentrations of the chitin synthase inhibitor polyoxin D, when compared to the wild type. In addition, the expression of chitin synthase genes was highly elevated in the *∆mas-1* strain, suggesting the gene product is involved in cell wall biosynthesis and the novel anhydride interferes with its function.

## 1. Introduction

An increasing number of reports on the association of fungi with sessile marine animals is accumulating [1]. Marine sponges have been shown to harbor diverse fungal communities that, in many cases, include ubiquitous species also found in terrestrial habitats [2]. A growing array of secondary metabolites produced by these fungi is considered a part of the complex relationship between marine sponges and their associate fungal communities [3]. These metabolites have been suggested to be involved in different ecological processes, including chemical defense of their sessile hosts [4,5,6] and defense against competitors [7]*.* Although the nature of the associations between sponges and fungi is far from understood, there is accumulating evidence that some sponge-associated fungi have adopted the ability to produce chemical compounds which are structurally diverged from their terrestrial counterparts. These compounds are considered a part of the chemical arsenal which enables niche specialization. In addition, a growing number of studies have indicated that sponge-derived fungi can provide sources of novel bioactive secondary metabolites, exhibiting anticancer, antibacterial, antiviral, anti-inflammatory, antifouling and antifungal agent properties [8,9]. Secondary metabolites in marine fungi are chemically diverse and are, among others, comprised of unusual nucleosides [10], terpenes [11], peptides [12], alkaloids [13], nonribosomal peptides [14] and polyketides [15]. Many studies have reported on the isolation of marine sponge-associated *Aspergillus* spp. as producers of bioactive metabolites [2]. Examples include a strain of the coral pathogen *A. sydowii* isolated from *Spongia obscura* [16] and *A. ustus*, isolated from the marine sponge *Suberites domuncula* that was found to produce seven new drimane sesquiterpenoids which showed cytotoxic activity against a panel of tumor cell lines [17]. Varoglu and Crews [18] isolated the new asperic acid from a saltwater culture of *A. niger* derived from a Caribbean sponge *Hyrtios proteus*. Chu *et al*. [19] described the biological active metabolite cyclo (l-Trp-l-Phe) produced by the fungus *A.* *versicolor* associated with the South China Sea sponge *Holoxea* sp. and Cohen *et al*. [20] described novel terpenoids from a Mediterranean *Psammocinia* sp.-associated *A. insuetus*. Although many studies describe novel compounds produced by sponge-associated fungi, the understanding concerning the biological activity and the mode of action of those metabolites is, in many cases, lacking. This is especially so in the case of compounds exhibiting antifungal properties.

The fungal cell wall is comprised of a mix of cross-linked fibers (mainly the polysaccharides glucan and chitin) and matrix components, primarily proteins and mannans. In filamentous fungi, growth and cell wall assembly occur mainly at hyphal apices, where the carbohydrate polymers are synthesized by membrane-associated enzymes, some of which are transported within vesicles to their site of activity. The polymers are then cross-linked and modified by extracellular proteins [21]. The main component of the primary cell wall is chitin, a β-1,4 polymer of *N*-acetylglucosamine and it is synthesized by chitin synthases. The bulk of the fungal cell wall is comprised of β-1,3 glucan and is synthesized by glucan synthases. The requirement of a functional cell wall for survival, growth, development and pathogenicity of fungal species makes it an attractive target for antifungals, especially due to the fact that some of the constituents of the fungal cell wall are not present in potential hosts [22]. Examples of fungal cell wall biosynthesis inhibitors include the peptide nucleoside antibiotics (polyoxins and nikkomycins) used to inhibit chitin synthases [23,24] as well as glycolipid papulacandins [25,26] and lipopeptide echinocandins [27] which block glucan synthesis. 

In this study, we report on a novel metabolite, **1**, produced by an *A. tubingensis* strain isolated from the sponge *Ircinia variabilis*. This metabolite exhibits anti-fungal activity, resulting in the impairment of cell wall integrity. Furthermore, we have determined that sensitivity to this compound can be conferred by a single novel protein in *Neurospora crassa* designated MAS1. Additional metabolites, the known 2-carboxymethyl-3-hexylmaleic acid anhydride [3], asperic acid [18], and campyrone A and C [28], were purified and their structures elucidated.

## 2. Results and Discussion

### 2.1. A Marine-Derived Aspergillus tubingensis Produces Novel Antifungal Compounds

Paz *et al*. [29], identified 25 *Eurotiales* (*Aspergillus* and *Penicillium* spp.) associated with the marine sponge *Ircinia variabilis*. We further analyzed strain OY907 and based on partial sequence of the *β-tubulin* gene used for identification of *Aspergillus* species [30], we have now identified it as *Aspergillus tubingensis* (Accession number KM079500).

*Aspergillus* species are widespread among marine sponges and usually constitute a large number of the total fungi isolated from many of these organisms [31]. Holler *et al*. [2] found that out of more than 680 fungal strains isolated worldwide from 16 sponge species, the majority belonged to the genera *Aspergillus* and *Penicillium*. The high occurrence of these genera was also evident in the report by Paz *et al.* [29] stating that 25 from a total of 80 Ascomycota taxa isolated from a Mediterranean sponge were *Aspergillus* and *Penicillium* spp. Similarly, among culturable fungi from two South China Sea sponges (*Clathrina luteoculcitella* and *Holoxea* sp.), these genera were also highly prevalent [32]. Although the genus *Aspergillus* has been widely investigated, to the best of our knowledge this is the first report of an *A.* *tubingensis* that has been isolated from a marine environment.

The specific *A. tubingensis* strain studied here (OY907) was found to secrete chemicals that inhibited the growth of three test fungi: *Alternaria alternata*,* Rhizoctonia solani* and *Neurospora crassa*, as determined by dual culture diffusion assays (inhibition zones wider than 15 mm from the source in the assay Petri dish). The former are Ascomycete and Basidiomycete plant pathogens, respectively, and the latter is a model Ascomycete which is highly amenable to genetic analysis and manipulation. 

**Figure 1:**
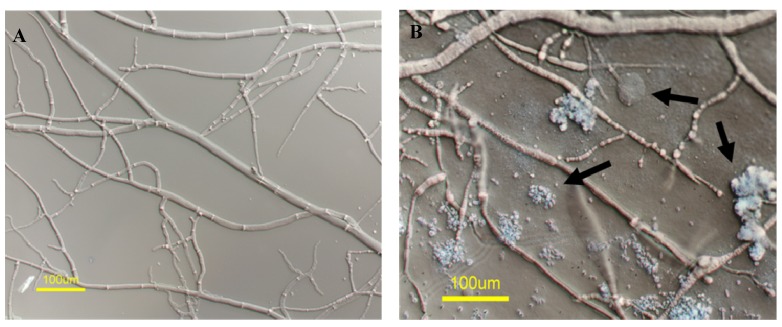
The effects of *Aspergillus tubingenis* crude extract amended on hyphal morphology of *Neurospora crassa*. Wild type cultured on (**A**) non-amended and (**B**) extract-amended (2 mg/mL) media, respectively. Arrows mark hyphal tip swelling, foci of cytoplasmic leakage and amorphous extracellular material.

We examined, in more detail, the morphological effects of the extract secreted by strain OY907 on *N. crassa*. When grown in the presence of 2 mg/mL of the crude extract, hyphae of *N. crassa* exhibited swollen tips with multiple foci of cytoplasmic leakage. Furthermore, significant amounts of amorphous material accumulated along the hyphae (Figure 1A,B).

A similar phenotype was observed in *A. alternata*, but not in* R. solani* (where the inhibition of radial growth was not accompanied by this morphological defect). As the outcome of exposure to the extract could involve more than one compound, fractionation and subsequent structure elucidation of antifungal compounds produced by this strain were carried out.

**Figure 2:**
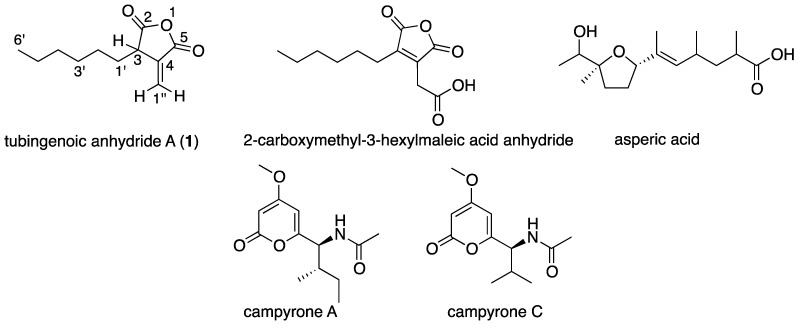
The metabolites isolated from *A. tubingensis* strain OY907.

Tubingenoic anhydride A (**1**; Figure 2) was isolated as a colorless oil that presented a negative HRESIMS pseudo-molecular ion at *m/z* 195.1024 corresponding to the molecular formula C_11_H_16_O_3_ and four degrees of unsaturation. Its ^1^H NMR spectrum (CDCl_3_ + CD_3_OD) presented two singlet protons at 6.34 and 5.72 ppm, a triplet proton at 3.40 ppm, two protons at 1.83 and 1.62 ppm, a 8H-multiplet at 1.24 ppm and a triplet methyl at 0.82 ppm. Based on the HSQC experiment, the ^13^C NMR spectrum of **1** presented four sp2 carbons (176.0 s, 168.7 s, 138.5 s and 127.2 t), a methine carbon (46.6 ppm), five methylene carbons (30.8, 31.4, 29.0, 27.3 and 22.4 ppm) and a methyl carbon (13.8 ppm). Correlations from the COSY experiment allowed the connection of the methine H-3 (3.40 ppm) with the protons of the diastereotopic methylene-1′ (1.82 and 1.61 ppm) and of the later with the eight-proton multiplet at 1.24 ppm. This multiplet was further correlated with the triplet methyl suggesting that a normal hexyl was attached to methine-3. This was supported by the HMBC correlations summarized in Table 1. Additional long-range COSY correlation of H-3 with the singlet proton at 5.71 ppm and of the later with the singlet proton at 6.34 ppm, both belonging to a terminal methylene (1″) based on the HSQC spectrum (Table 1), suggested that H-3 is allylic to the terminal methylene. The chemical shift of the terminal methylene protons, suggested their conjugation to a carbonyl as was evident from their strong (^3^*J*) HMBC correlations with the carbon resonating at 168.7 ppm (C-5). The terminal methylene protons exhibited additional HMBC correlations (^2^*J*) with a quaternary olefin carbon (138.5 ppm, C-4), strong correlations (^3^*J*) with C-3 and weak correlations (^4^*J*) with the sp^2^ carbon at (176.0 ppm). H-3 exhibited correlations with all four sp^2^ carbons and two methylene carbons resonating at 30.8 (1′) and 27.3 ppm suggesting that it was bridging between the aliphatic and unsaturated moieties of **1**. The diastereotopic protons of methylene-1′ exhibited HMBC correlations with the two quaternary sp^2^ carbons resonating at 176.0 and 138.5 ppm, with methine-3 and with two methylene carbons resonating at 27.3 and 29.0 ppm. These correlations established the attachment of the hexyl moiety to methine-3, the vicinity of methylene-1′ to the carboxyl carbon resonating at 176.0 ppm and to the quaternary olefin carbon (138.5 ppm), and assigned the methylene resonating at 27.3 and 29.0 ppm as 2′ and 3′, respectively. The correlations of the methyl protons with the methylene carbons resonating at 31.4 and 22.4 ppm, assigned them as C-4′ and C-5′, respectively. Based on the molecular formula inferred from the HR mass measurements the two carboxyls of **1** are engaged in an anhydride linkage. Based on the arguments summarized above the structure of tubingenoic anhydride A (**1**) was established as 3-hexyl-4-methylenedihydrofuran-2,5-dione.

**Table 1:** NMR Data of Tubingenoic Anhydride A (**1**) in CDCl_3_ + CD_3_OD ^a^.

Position	δ_C_, Multiplicity	δ_H_, Multiplicity	HMBC Correlations
2	176.0, qC		H-3, H-1′a, H-1′b, H-1″a, H-1″b
3	46.6, CH	3.40 t, 7.4	H-1′a, H-1′b, H-1″a, H-1″b, H_2_-2′
4	138.5, qC		H-3, H-1′a, H-1′b, H-1″a, H-1″b
5	168.7, qC		H-3, H-1″a, H-1″b
1′	30.8, CH_2_	a 1.82, ddt b 1.61, ddt	H-3, H_2_-2′, H_2_-3′
2′	27.3, CH_2_	1.25, m	H-3, H-1′a, H-1′b, H_2_-3′
3′	29.0, CH_2_	1.22, m	H-1′a, H-1′b, H_2_-2′
4′	31.4, CH_2_	1.22, m	H_2_-2′, H_3_-6′
5′	22.4, CH_2_	1.20, m	H_2_-3′, H_3_-6′
6′	13.8, CH_3_	0.82 t, 6.7	H_2_-4′
1″	127.2, CH_2_	a 5.71, s b 6.34, s	H-3

^a^ 500 MHz for ^1^H and 125 MHz for ^13^C.

Tubingenoic anhydride A (**1**) is a polyketide metabolite exhibiting anti-fungal activity (*N. crassa* MIC = 330 μM) derived from the conjugation of octanoic acid and C_4_ metabolite, probably oxaloacetic acid and decarboxylation. Related metabolites were previously isolated from several *Aspergillus* spp. 2-Carboxymethyl-3-hexylmaleic acid anhydride was isolated from *Aspergillus* *awamori* as a weak antibiotic of gram-positive bacteria [33]. We found this compound to also exhibit an inhibitory effect on *N. crassa* (MIC = 207 μM). 2-Carboxymethyl-3-hexylmaleic acid anhydride and 2-methylene-3-hexylbutanedioic acid were isolated from *A. niger* as enhancers of seed germination, and shoot and root growth in cauliflower seedlings [34]. Related metabolites were characterized from other fungi, lichen [35] and plants [36]. The fact that compounds related to the metabolites described here affect the growth of a variety of organisms may have implications concerning the compounds described here. The growth promotion and/or inhibitory effects these compounds may have on the microbiota of the sponge, or the sponge itself, may prove to have ecological significance if they are also produced in the sponge holobiont.

### 2.2. mas-1 Is a Novel N. crassa Gene Conferring Sensitivity to Antifungal Compounds Produced by A. tubingensis Strain OY907

**Figure 3:**
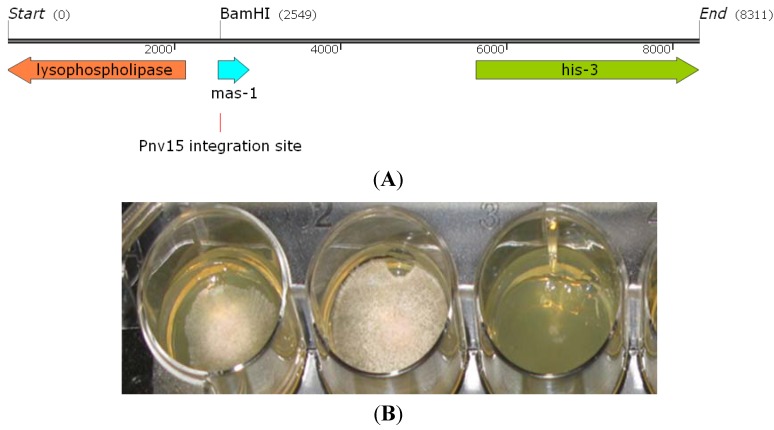
Disruption of *mas-1* confers tolerance of *Neurospora crassa* to metabolites produced by *Aspergillus tubingensis*. The *mas-1* locus and Pnv15 integration site (**A**). m-42, *mas-1* and wild type strains grown in medium amended with the crude extract produced by *A. tubingensis* (**B**).

In order to provide insight on the mechanism(s) involved in conferring sensitivity of *N. crassa* to the secreted antifungal metabolites produced by strain OY907, a genetic approach was employed. The wild type strain was subjected to tagged insertional mutagenesis with the use of plasmid pNV15 which harbors a Nourseothricin (NAT) resistance cassette. The Nat^R^ transformants were examined for resistance to the antifungal compounds. Analysis of progeny from purified homokaryons crossed to the wild type confirmed the genetic link between Nat^R^ and the ability to grow on the crude extract. One of the transformants (m-42) was used for further analysis. To determine the integration site of the pNV15 construct, DNA contiguous with the inserted plasmid was retrieved by a plasmid rescue procedure. Genomic DNA of the resistant mutant was digested with *Bam*HI (which does not cleave within the mutagenesis plasmid) and the ligated product was sequenced. DNA sequence flanking the pNV15 insert was then used to probe the *N. crassa* genome database [37] The disrupted locus was found to be a new gene (NCU03140) that encodes for a yet uncharacterized, small (91 aa), hypothetical protein (Figure 3A) whose disruption does not result in any observable defect under standard growth conditions. Since there were no homologs to this gene in other fungi or indications for the presence of a conserved motif in the NCBI databases, we designated this new gene *mas-1* (Marine-Aspergillus Sensitive 1). Even though the genome of several hundred fungal strains has been sequenced, many genes in the highly diverse members of this kingdom have not been assigned functions. This is also true for *N. crassa*, the first published full genome of a filamentous fungus [37,38], where hundreds of genes have yet to be assigned functions. 

As *mas-1* is a unique, yet uncharacterized gene, several measures were taken in order to fully confirm the relevance of *mas-1* to the sensitivity of *N. crassa* to the *A. tubingensis* metabolites. To verify that no annotation error (resulting in miscall of the small *mas-1* gene) had occurred during the analysis of the *N. crassa* genome, we examined the *∆mas-1* (NCU03140) deletion strain obtained from the FGSC and compared it to strain m-42. As both strains were resistant to the antifungal extract, we concluded that *mas-1* is, indeed, involved in conferring the biological activity of the *A. tubingensis* metabolites to *N. crassa* (Figure 3B). In additional experiments, when tested on medium amended with the purified novel tubingenoic anhydride A (**1**), the wild type was highly sensitive to the metabolite compared to the ∆*mas-1* strain, at a concentration of 0.8 mg/mL. 

**Figure 4:**
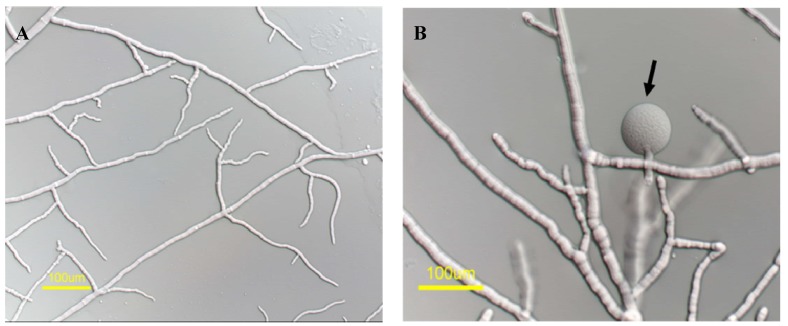
Δ*mas-1* cultured on (**A**) non-amended and (**B**) extract-amended (2 mg/mL) media. Arrow marks hyphal tip swelling.

When conidia of the *∆mas-1* strain were placed on medium amended with the *A. tubingensis* extract, they germinated to produce normal appearing hyphae, with the exception of a few cases of hyphal swelling and cytoplasmic leakage. Hence, even though the absence of *mas-1* confers a high degree of tolerance to the crude extract, at concentrations of 2 mg/mL or higher, some effects of the antifungal compound could still be observed (Figure 4), indicating that *mas-1* is not the only gene involved in conferring the effects of the antifungal compound. Otherwise, deletion of *mas-1* did not affect the fitness of *N. crassa* in terms of growth rate and amount and types of conidia (data not shown).

**Figure 5:**
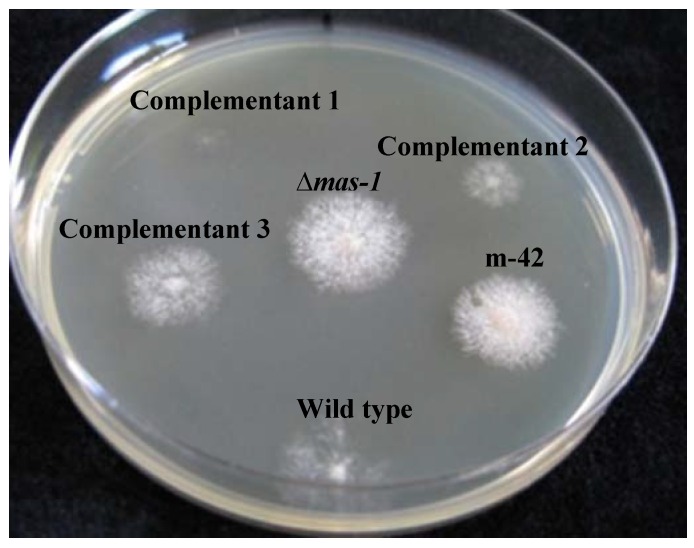
Re-introduction of *mas-1* restores *N. crassa* sensitivity to the antifungal compounds produced by* A. tubingesis*. Comparative growth of the wild type, m-42 mutant, ∆*mas-1* and three independent m-42 mutant transformants which harbor the wild type allele of *mas-1* grow on media amended with a sublethal concentration (1 mg/mL) of crude extract.

As *mas-1* is a small gene and is closely linked to two additional genes: Histidine biosynthesis trifunctional protein (*his-3*; NCU03139) and Lysophospholipase (NCU03141) (Figure 4A), that may have been affected by the mutagenesis procedure (in either or both resistant strains), we performed genetic complementation by introducing a wild-type copy of *mas-1* into the m-42 mutant. To do so, we transformed the mutant with pLK2, containing a wild type copy of the *mas-1* gene and cotransformed the m-42 strain along with a pCSN44 plasmid that contained the hygromycin B resistance gene cassette as a dominant selectable marker [39]. Hyg^R^ transformants were tested for their sensitivity to the *A. tubingensis* crude extract. All transformants tested regained sensitivity to the crude extract (albeit at different levels, possibly due to the locus of pLK2 integration) (Figure 5). Taken together, our results confirmed that an intact *mas-1* does, indeed, confer sensitivity of *N. crassa* to the crude extract produced by the marine-derived *A. tubingensis.*

### 2.3. Lack of mas-1 Increases the Resistance of N. crassa to Polyoxin D

**Figure 6:**
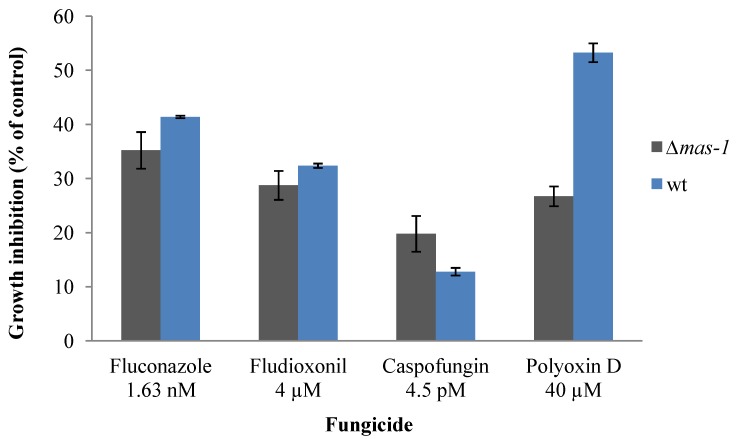
Effect of sub-lethal concentrations of four antifungal compounds on radial growth of the wild type and the Δ*mas-1* strains.

As the nature of the phenotypic consequences in the presence of the *A. tubingensis* extract indicated that hyphal integrity may be compromised, we analyzed the sensitivity of the ∆*mas-1* strain to several fungicides whose function results in impaired hyphal integrity. Sublethal concentrations of Fluconazole (an ergosterol biosynthesis inhibitor) or fludioxonil (an activator of the hyperosmotic stress response pathway) did not differentially affect the growth of *mas-1* when compared to the wild type (Figure 6). However, when grown in the presence of the chitin synthase inhibitor polyoxin D, the ∆*mas-1* strain was only about half as sensitive when compared to the wild type (26% and 53% inhibition of radial growth in the presence of 40 μM of the drug, respectively). These data suggest that the chitin constituent of the cell wall may be compromised in a ∆*mas-1* background. Furthermore, the fact that the increased tolerance of the ∆*mas-1* strain to Polyoxin D was not accompanied by a similar trend when exposed to the glucan synthesis inhibitor caspofungin, suggests that the functional link of MAS1 to chitin biosynthesis is specific. Alternatively, the lack of MAS1 may also affect chitin synthesis in an indirect manner, e.g., via altering the capacity of the fungus to uptake the chitin synthase inhibitor. Regardless of the precise mechanism involved, the interest in all aspects of fungal cell wall integrity as a potential target for antifungals [40,41] warrants additional functional analysis of the novel compound described as well as the role *mas-1* (or functional counterparts in other fungi) play in maintenance of fungal cell integrity. The cross-species sensitivity and the similar defects in the test fungi analyzed on the one hand, and the fact that *mas-1* is a unique gene, on the other, suggest that other genes in *A. alternata* and *R. solani* (and likely in *N. crassa* as well) are involved in conferring sensitivity/tolerance to the newly identified compound. Analysis of addition *N. crassa* mutants tolerant to the compound as well as further analysis of the other test fungi may provide a better mechanistic understanding of the compounds’ mode of action. Such information has the potential to be utilized in the development of antifungal treatments based on cell wall impairment as a target.

### 2.4. Chitin Synthase Gene Expression Is Altered in Response to the A. tubingensis Extract

**Figure 7:**
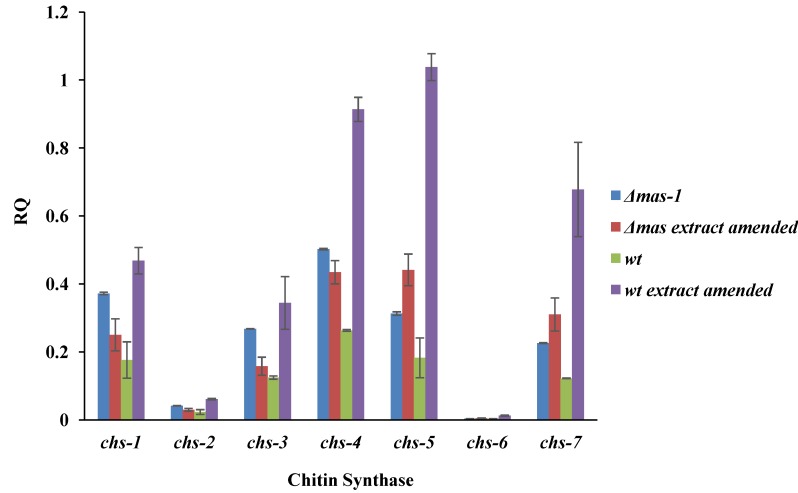
Relative expression levels of seven *chitin synthase* genes in *N.* *crassa* wild type and ∆*mas-1* grow on non-amended and extract-amended (1 mg/mL, a sub lethal concentration) media, as determined by Real Time PCR. RNA was extracted from conidial germlings and expression was normalized using the *beta tubulin* gene. Relative expression levels were calculated using the 2^−Δct^ method.

To provide additional insight as to the link between the *A. tubingensis* antifungal products and cell wall integrity, we examined the relative expression of the seven *chs* genes in the wild type and in ∆*mas-1.* When exposed to a sublethal concentration (1 mg/mL) of the *A. tubingensis* crude extract, a significant increase in the transcript levels of all *chs* genes was observed in the wild type strain (Figure 7), an expected cellular response to perturbation of cell wall integrity (with emphasis on the chitin component). The extent of that response was less pronounced in the ∆*mas-1* strain. However, *chs* genes expression in the mutant was approx. two-fold higher than in the wild type to begin with (in the absence of the extract). These higher expression levels include *chs-4*, a gene whose product has been implied to be an “auxiliary enzyme” under stressful conditions [42]. It is tempting to speculate that the lack of *mas-1* confers a precondition in which the fungal cell has an elevated pool of chitin synthases that contributes to its tolerance to the *A. tubingensis* products. Even though exposure to sublethal *A. tubingensis* extract concentrations results in increased *chs* expression in the wild type, the expression levels may not be sufficient to confer tolerance once the fungus has been exposed to the antifungal compounds. It is also possible that MAS1 functions in additional manners that increase tolerance, such as an effect on chitin synthase localization/activation or via additional components involved in cell wall integrity. Overall, these results indicate that the *A. tubingensis* crude extract has an effect on *chs* genes expression and that at least part of the changes in *chs* genes expression is mediated by MAS1. 

### 2.5. Cellular Localization of MAS1::GFP

**Figure 8:**
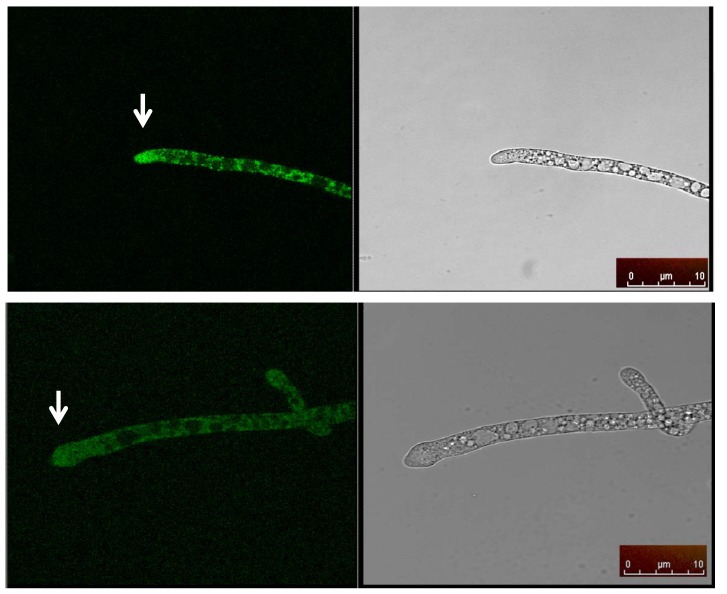
Confocal microscopy image of *N. crassa* hype expressing MAS1::GFP accumulated in small, round organelles especially at the hyphal apex (white arrows).

In order to determine the cellular localization of MAS1, we constructed a MAS1::GFP fusion (designated pLK4) driven by the *N. crassa* inducible *ccg-1* promoter pMF272 [43]. The ∆*mas-1* strain was co-transformed with pLK4 and pNV15 (conferring NAT^R^). The resulting transformants restored sensitivity to the crude extract, verifying the functionality of the MAS1::GFP fusion insert. Western blot analysis using anti-GFP antibodies was used to verify the presence of tagged MAS1. Confocal microscopy indicated that MAS1::GFP is distributed throughout the hyphal cell in a punctate manner, with marked presence of the protein in yet unidentified round organelles (Figure 8). These bodies accumulated especially at the hyphal apex. When grown on the presence of the *A. tubingensis* crude extract, no detectable change in MAS1::GFP localization was observed. Our results suggest that MAS1 is a cytosolic protein that accumulates (or is ferried) in specific cellular compartments. Furthermore, MAS1 was not detected in conidia. Though some chitin synthases are ferried in distinct vesicles [44], the pattern observed here was different from the distribution patterns characteristic of chitosomes [45]. 

In summary, marine sponge-associated fungi have a great potential to be a rich source of promising antifungal compounds, and this may be due, in part, to the unique ecological grow conditions (including the presence of additional, competing, fungi in this exceptional niche). Even though the compounds described have relatively high MICs, the potential of combining these or structurally related compounds with more effective antifungals (cell wall inhibitors or others) may provide an additional basis for antifungal intervention. The abundance of resources available for the analysis of *N. crassa* provides an attractive platform for the analysis of the mode of action of novel compounds as a step in the development of antifungal strategies.

## 3. Experimental Section 

### 3.1. General Experimental Procedures—Chemical Analysis

Optical rotation values were obtained on a Jasco P-1010 polarimeter at the sodium D line (589 nm). UV spectra were recorded on an Agilent 8453 spectrophotometer. High-resolution MS were recorded on a Waters MALDI Synapt instrument. NMR spectra were recorded, at 25 °C, on a Bruker DRX-500 spectrometer at 500.13 MHz for ^1^H and 125.76 MHz for ^13^C. COSY-45, gTOCSY, gROESY, gHSQC and gHMBC spectra were recorded using standard Bruker pulse sequences. HPLC separations were performed on a Jasco HPLC system (model PU-2080-plus pump, model LG-2080-04 Quaternary gradient unit and model MD-2010-plus Multi-wavelenght detector) and on an Agilent 1100 series HPLC system. 

### 3.2. Fungal Strains, Media, and Growth Conditions

An *Aspergillus* sp. strain (OY907) previously isolated from the Mediterranean sponge* Ircinia variabilis* (formerly designated as a *Psammocinia* sp. Paz *et al*. [29]) was cultured either on Potato Dextrose Agar (PDA; Difco) or mass cultivated in 1 L stationary flasks containing 200 mL Potato Dextrose Broth (PDB; Difco) for 25 days at 27 °C in the dark. *Alternaria alternata* (Aa01) and *Rhizoctonia solani* (TP6) form the Department of Plant Pathology and Microbiology collection were maintained on PDA. N. crassa strains used in this study were the wild type (74-OR23-1A), a Δ*mas-1* *N. crassa* knock out mutant strain (FGSC14039; NCU03140), as well as *chs-1*, -*3*,-*4*,-*5*,-*6* and *7*, which were obtained from the Fungal Genetic Stock Center. The *chs-2*^RIP^ strain was described in a previous study [46]. *N. crassa* strains were grown in either liquid or solid (supplemented with 1.5% agar) Vogel’s minimal medium supplemented with 1.5% (w/v) sucrose (Vs). Unless stated otherwise, all growth and manipulations of *N. crassa* were as previously described [47]. When required, the medium was supplemented with 100 μg/mL hygromycin B (Calbiochem, Riverside, CA) or 20 μg/mL nourseothricin (Werner Bioagents, Jena, Germany). Fungicides used in this study were: fluconazole (Orius, Makhteshim-Agan) which is an ergosterol biosynthesis inhibitor, fludioxonil (Fluka analytical, Sigma Aldrich) an activator of the hyperosotic stress response pathway, caspofungin, a (1→3)-β-d-glucan synthase inhibitor (Merck Research Laboratories, Rahway, NJ, USA) and polyoxin D (Kaken Chemical Company, Tokyo, Japan) a chitin synthase inhibitor. For growth rate measurements, 10 μL of a conidial suspension (1 × 10^7^ conidia/mL) were inoculated on Petri dishes or race tubes containing Vs and growth was measured periodically.

### 3.3. Antifungal Activity Assays

To determine the presence of secreted anti-fungal compounds, an agar medium diffusion assay was performed using* N. crassa*, *A. alternata* and *R. solani* [48]. A mycelial disk of strain OY907 was placed in the center of a petri dish containing PDA and disks of the test fungi were placed on the Petri dish periphery. Following 3 or 14 days of incubation (to test the effects on *N. crassa* and the other two fungi, respectively), the effect of diffused compounds from the marine isolate was assessed. The formation of inhibition zones wider than 15 mm between the OY907 source and the test organisms was considered as an indicator for the presence of inhibitory compounds. 

### 3.4. Chemical Analysis of the Antifungal Product

Strain OY907 was mass cultivated on PDB in stationary flasks for 25 days at 27 °C in the dark**. **The mycelium was separated from the growth medium by filtration and then extracted two times with ethyl acetate 1:1 (v:v). Ethyl acetate extracts were combined, filtered and evaporated. The dry crude extract was dissolved in dimethylsulfoxide (DMSO) and tested for its antifungal activity. The extract was fractionated on a reversed-phase open column followed by gel-filtration on Sephadex LH-20. Each fraction was tested for biological activity by amending the *N. crassa* growth medium with a range of concentrations (0.5–2 mg/mL) of the dried fraction. Final purification of the active (antifungal) compounds was achieved by HPLC separation. The active fraction was retested for antifungal activity and the structure of the purified compound was elucidated by1D and 2D NMR technique and HR mass spectrometry.

Tubingenoic anhydride A (**1**). 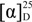 −6.3 (*c* 0.02, MeOH); UV (MeOH) λ_max_ (log ɛ) 202 (3.58); IR (CHCl_3_) ν_max_ 3020, 2929, 2858, 1708, 1629, 1522,1421,1212 cm^−1^; For NMR data see Table 1; HR ESI MS *m/z* 195.1024 ([M − H]^−^, calcd. for C_11_H_15_O_3_* m/z* 195.1021).

### 3.5. Disruption of mas-1

In order to study the function of the *A. tubingensis* secreted metabolites, a *N. crassa* resistance mutant was produced by random tagged insertional mutagenesis by electroporation [49]. We used plasmid pNV15 which harbors a Nourseothricin (NAT) resistance cassette [50] that linearized by *Sac*I and subjected to wild type. The inserted plasmid was retrieved following a plasmid rescue procedure [51]. Genomic DNA of the resistant mutant was digested with *Bam*HI. The digested products were subsequently subjected to ligation (T4 ligase Thermo scientific) in a 100 µL reaction volume. The ligated products were then cloned into *E. coli* HIT DH5α competent cells (RBC Bioscience) and resulting clones were sequenced.

### 3.6. Functional Complementation

For introducing a wild-type copy of *mas-1* into the m42 mutant, the *mas-1* gene was amplified by using primers *f-1200* and *r-609* that include the *mas-1* gene as well as 1200 bp and 609 bp of DNA upstream and downstream of the coding region, respectively. The PCR product was cloned into a pDRIVE expression vector (QIAGEN) according to the manufacturer’s instructions and designated pLK3. pLK3 was restricted with *Stu*I and the linear construct was cotransformed into the *N. crassa* m-42 strain along with pCSN44 (which contains a hygromycin B resistance cassette) [39]. The resulting transformants were tested for their ability to grow on hygromycin B and for sensitivity to the crude extract from the *A. tubingensis* growth medium. PCR amplification of the inserted cassette utilizing primers *hphf* and *hphr* and primers *mas-1f* and *mas-1r* (Table 2), were used to verify insertion of the pLK3 plasmid and the pCSN44 plasmid into m-42 mutant.

### 3.7. Chitin Synthase Gene Expression

*N. crassa* has been shown to have seven genes encoding chitin synthases [38]. In order to determine whether deletion of *mas-1* conferred changes in the expression of the different chitin synthase (*chs*) genes, we preformed Real Time PCR for all the seven *chs* genes. Since genes encoding for CHS in *N. crassa* are highly similar, manual primer design was carried out in order to identify unique 80–150 bp amplicons. Confirmation of primer set specificity was performed by Real Time PCR on cDNA templates of the mutants lacking genes encoding for CHS.

For determining *chs* gene expression, RNA was extracted from conidial germlings (from the wild type and ∆*mas-1* strains) that were grown on liquid Vs amended with 1 mg/mL (a sub lethal concentration) of the crude extract for 4 h at 34 °C at 150 rpm. The RNA samples were purified with the RNeasy Plant Mini Kit (Qiagen) as previously described [47]. Purified RNA (1 μg) was used for the RT procedures using SuperScript II RNase H reverse transcriptase (Invitrogen, Carlsbad, CA, USA) according to the manufacturer’s instructions. Relative quantification of transcript abundance of the 7 *chs* genes was performed on an ABI StepOnePlus Real-Time PCR Sequence Detection System and software (Applied Biosystems) as described [52]. RT-PCR mixtures were comprised of a 3.3 µM concentration of each primer (see Table 2), 5 μL of SYBR green PCR master mix (Applied Biosystems), 2 μL of cDNA at concentration of 10 ng, and nuclease-free water to a final volume of 10 μL. The annealing temperature was modified to 64 °C, utilizing *chs 1-7* primers (see Table 2). Total cDNA abundance in the samples was normalized using the *beta tubulin* gene (NCU04173.3). In all experiments, samples were amplified in triplicate, and the average cycle threshold was then calculated and used to determine the relative expression of each gene. Relative expression levels were calculated using the 2^−Δct^ method [53].

**Table 2:** Primers used in this study.

Name	Sequence
*f-1200*	CCAGTTCTTCTCCTCGTTCG
*r-609*	TTCGTTCGCTGTCAATCAA
*hphf*	GTCGGAGACAGAAGATGATATTGAAGGAGC
*hphr*	GTTGGAGATTTCAGTAACGTTAAGTGGAT
*mas-1f*	ATGGCAAGGGCAAGAATTG
*mas-1r*	TCAGTGGTTGTGCCATTCAG
*gfp-1f*	AAGGTCTAGAATGGCAAGGG
*gfp-1r*	CCCGGGGTGGTTGTGCCATT
*gfp-2f*	GACGTAAACGGCCACAAGTT
*gfp-2r*	GAACTCCAGCAGGACCATGT
*nat-1f*	GGGCAGTAAGCGAAGGAGAATG
*nat-1r*	GGGATGGGAAGGATGGAGTA
*chs-1f*	GTGGGGTACCAAGGGTTCGG
*chs-1r*	CCTGTGGCTTCTCAATCTCT
*chs-2f*	TCTGGACAGCGACCTCAAGTTCAA
*chs2-r*	TGCCAAAGGCGTTGAAGAACCATC
*chs-3f*	TCAAGAACGATGTCGTCCAGCTCA
*chs-3r*	CAAAGGCCTGGAAGAACCAACGAT
*chs-4f*	TCTGGACTCGATTGCAATGACGGA
*chs-4r*	TTCCTCTCCGTGGCCCTTGATAAT
*chs-5f*	AAATCTCGGGCTTCTCATACGCCA
*chs-5r*	AACGGGCAGATGTGTCTTATCGCT
*chs-6f*	AAGACGGGTGACGACCTCAA
*chs-6r*	TAATGCCGGTGGTGAAGCCC
*chs-7f*	ACCCTCAACCTTACAGGCAACCTT
*chs-7r*	CAACAAGCCTTTGTCGGTGTCGAT

### 3.8. MAS1::GFP Fusion Protein Construct and Confocal Imaging

In order to monitor the protein *in vivo* for cellular localization, MAS1 was tagged with a green fluorescent protein (GFP). The putative *mas-1* ORF (381 bp) was amplified, without the stop codon, as an *Xma*I/*Xba*I fragment (390 bp) utilizing primers gfp-1f and gfp-1r (Table 2) and cloned into a pDrive vector (Qiagen) to yield pLK1. The pLK1 insert, retrieved following *Xma*I/*Xba*I restriction and gel purification, was ligated into an *Xma*I/*Xba*I digested pMF272, a *sgfp* plasmid driven by the *N. crassa* *ccg-1* promoter (AY598428) [43] which is induced by glucose deprivation or stress [54]. This yielded pLK2, an expression plasmid used for MAS1 localization and for complementation of the Δ*mas-1 strain*. pLK2 was digested with *Sap*I/*Pst*I and the gel-purified fragment (4 kb) was used to cotransform the mutant strain along with *pNV15*. The transformed isolates were selected for their ability to grow on NAT. PCR amplification and subsequent sequencing of pLK2 utilizing primers *gfp-2f* and *gfp-2r* (Table 2), were performed to verify insertion of the pLK2 plasmid into Δ*mas-1*. In addition, primers *nat-1f* and *nat-1r* (Table 2) were used to verify the presence of the pNV15 insert. Transformant homokaryons were purified by isolation of microconidia. The GFP-expressing strains were first grown on Vogel’s medium with 2% glucose for 18 h at 34 °C, then, harvested, washed with water and resuspended in Vogel’s minimal medium with no carbon source for an additional 2 h. The presence of the MAS1::GFP fusion protein was determined by western blot analysis as described [55]. Primary antibodies used were mouse anti-GFP (Covance, Princeton, NJ, USA) followed by goat anti-mouse secondary antibodies (Amersham Biosciences, Freiburg, Germany). For cellular localization, confocal laser scanning inverted microscopy was performed using a LEICA TCS SP8 system equipped with an argon ion laser. The strains were imaged by excitation with the 488 nm laser line and emission at 510–530 nm. Oil immersion 60× or dry 20× lenses were used for imaging.

## 4. Conclusions

In this study, we have identified a fungal strain previously isolated from the Mediterranean marine sponge *Ircinia variabilis* as *Aspergillus* *tubingensis*. We have purified and identified a novel compound that exhibits antifungal properties and have identified it as Tubingenoic anhydride A. We have determined that a small (91 aa), yet uncharacterized protein, MAS1, confers sensitivity to the antifungal products in* N. crassa*. The Δ*mas-1* strain was two-fold more resistant to the chitin synthase inhibitor Polyoxin D than the wild type, suggesting, that the *mas-1* gene is involved in the cell wall integrity. Moreover, the constitutive elevated expression of chitin synthase genes in the Δ*mas-1* mutant supports the link of MAS1 to cell wall integrity. 
